# Graph Analysis and Modularity of Brain Functional Connectivity Networks: Searching for the Optimal Threshold

**DOI:** 10.3389/fnins.2017.00441

**Published:** 2017-08-03

**Authors:** Cécile Bordier, Carlo Nicolini, Angelo Bifone

**Affiliations:** Center for Neuroscience and Cognitive Systems, Istituto Italiano di Tecnologia Rovereto, Italy

**Keywords:** threshold, percolation, sparsification, brain networks, functional connectivity

## Abstract

Neuroimaging data can be represented as networks of nodes and edges that capture the topological organization of the brain connectivity. Graph theory provides a general and powerful framework to study these networks and their structure at various scales. By way of example, community detection methods have been widely applied to investigate the modular structure of many natural networks, including brain functional connectivity networks. Sparsification procedures are often applied to remove the weakest edges, which are the most affected by experimental noise, and to reduce the density of the graph, thus making it theoretically and computationally more tractable. However, weak links may also contain significant structural information, and procedures to identify the optimal tradeoff are the subject of active research. Here, we explore the use of percolation analysis, a method grounded in statistical physics, to identify the optimal sparsification threshold for community detection in brain connectivity networks. By using synthetic networks endowed with a ground-truth modular structure and realistic topological features typical of human brain functional connectivity networks, we show that percolation analysis can be applied to identify the optimal sparsification threshold that maximizes information on the networks' community structure. We validate this approach using three different community detection methods widely applied to the analysis of brain connectivity networks: Newman's modularity, InfoMap and Asymptotical Surprise. Importantly, we test the effects of noise and data variability, which are critical factors to determine the optimal threshold. This data-driven method should prove particularly useful in the analysis of the community structure of brain networks in populations characterized by different connectivity strengths, such as patients and controls.

## Introduction

In recent years, considerable efforts have been made to study the complex structure of brain connectivity, marking the inception of the “connectomic era” in brain neuroscience. Functional Magnetic Resonance Imaging (fMRI) and other neuroimaging methods have shown that spontaneous fluctuation in brain activity, as measured with a subject lying in the scanner without being engaged in any specific task, are organized in coherent patterns, thus suggesting that resting state functional connectivity reflects the functional architecture of the brain (Damoiseaux et al., 2006).

Several methods have been developed and applied to study these patterns of synchronization, including multivariate approaches (e.g., Principal Component or Independent Component Analysis) (Beckmann et al., 2005; Damoiseaux et al., 2006) and graph theoretical methods (Bullmore and Sporns, 2009).

Graph theory provides a general and powerful framework to investigate the topological organization of the brain connectivity. A number of graph theoretical studies have revealed a small-world, rich-club structure (van den Heuvel and Sporns, 2011) of functional connectivity networks, and the presence of hub regions defined by high connectivity and network centrality. Moreover, community detection methods have been widely applied to investigate the modular structure of many natural networks, including brain functional connectivity networks. The presence modules, i.e., clusters of nodes that are more densely connected among them than with the rest of the network, reflects functional segregation within the integrated network, and is thought to confer robustness and adaptability to brain connectivity networks (Bullmore and Sporns, 2009).

For these analyses, the brain is represented as a network of nodes interconnected by links. Commonly, the nodes correspond to anatomically defined brain areas and links to a measure of inter-regional interaction or similarity between the nodes. For resting state functional connectivity networks, edge weights are typically computed as temporal correlations in the fluctuations of the BOLD signals in different areas, resulting in a correlation adjacency matrix (Eguiluz et al., 2005).

Sparsification procedures are normally applied to remove weaker links, which are most affected by experimental noise (van den Heuvel and Fornito, 2014), and to reduce the density of the graph, thus making it computationally more tractable. In the literature, it is common practice to fix the density of the adjacency matrix *a priori*, and to identify the threshold that preserves the target density of edges (Bassett et al., 2008; Lynall et al., 2010). Stability analyses exploring a range of densities are often performed to assess how critically topological parameters derived from the sparsified adjacency matrix depend on the choice of threshold. Sparsification schemes based on the computation of graph Minimum Spanning Trees prior to thresholding have also been proposed to prevent disruption of local connectivity by global removal of weak links (Alexander-Bloch et al., 2010).

Here, we address the problem of computing the optimal threshold for community detection in brain connectivity networks. Specifically, we propose the use of percolation analysis, a method rooted in statistical physics, to identify a sparsification threshold that maximizes information on the network modular structure. This data driven procedure, first introduced by Gallos et al. (2012), iteratively removes the weakest edges and computes the largest connected component. The percolation threshold corresponds to the point where the largest component starts breaking apart. We entertain the hypothesis that the percolation threshold strikes the optimal balance between information gained by cutting off noise, and lost by removing potentially genuine weak connections. To test this hypothesis, we apply three different community detection methods (Newman's modularity Newman, 2006), InfoMap Rosvall and Bergstrom, 2008; Kawamoto and Rosvall, 2015, and Asymptotical Surprise Nicolini and Bifone, 2016; Nicolini et al., 2017) to synthetic networks endowed with a ground truth modular structure, and with topological features, levels of noise and variability similar to those observed in functional connectivity experimental data. We compare the retrieved and planted modular structures by using Normalized Mutual Information, an information theoretic measure of similarity, as a function of sparsification threshold. We find that this information can be maximized by an appropriate choice of threshold, and we assess the use of percolation analysis as a data-driven method for optimal sparsification. Finally, we discuss the application of this approach to compare networks characterized by different noise levels and connectivity strengths, such as those observed in cross-sectional studies assessing brain connectivity in different populations, e.g., patients and healthy controls.

## Materials and methods

Synthetic networks are a useful tool to test the effect of threshold on community detections, and the ability to retrieve a pre-determined ground-truth modular structure. We ran two types of simulations: simulation of Lancichinetti-Fortunato-Radicchi (LFR) networks (Lancichinetti et al., 2008) and simulation of complex LFR including intersubject variability and different level of noise. The latter made it possible to assess the influence of noise or data variability, thus mimicking realistic experimental dataset.

The main goal of these simulations is the validation of a method that can be used in the analysis of functional connectivity networks as measured by resting state fMRI. As shown in Nicolini and Bifone (2016), brain functional connectivity networks are composed of modules with heterogeneous size distributions. This structure can be mimicked using the LFR approach, which can generate synthetic networks with power law degree distributions and community sizes akin to those observed in natural networks, such as functional connectivity networks (Lancichinetti et al., 2008).

### Simulation 1

**Figure 1:**
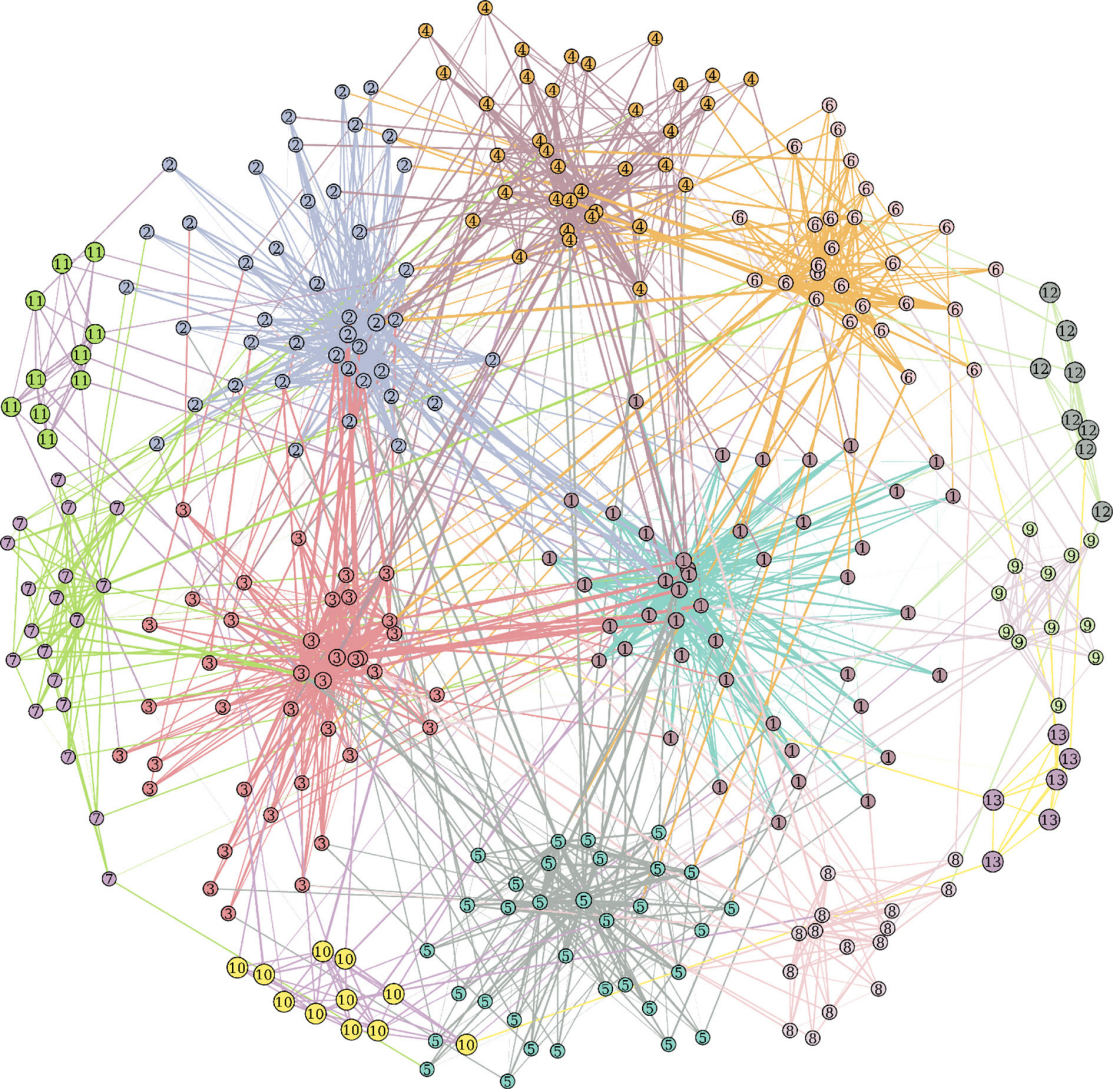
Example of benchmark LFR network with parameters *N* = 300, 〈k〉 = 12, max_k_ = 50, μ_t_ = μ_w_ = 0.2, min_c_ = 5, max_c_ = 50.

The Lancichinetti-Fortunato-Radicchi (LFR) benchmark algorithm generates networks with *a priori* known communities and node degree distributions. Community size and node degree follow power law distributions (for example see Figure 1).

The mixing of the communities is controlled by the topological mixing parameter μ_t_. Each node shares a fraction *1–*μ_*t*_ of edges with nodes in its same community and a fraction μ_t_ with nodes in other communities: *0* ≤ μ_*t*_ ≤ *1*. Similarly, a weight mixing coefficient μ_w_ controls, on average for each node, the balance between the incident edge weights coming from internal and external communities.

The LFR synthetic networks were built for *N* = 600 nodes, sampling nodes degree from a power-law with exponent τ_*d*_ = 2, average degree 〈k〉 = 12 and maximum degree max_k_ = 50. We set the topological and weight mixing coefficient, i.e., the average fraction of intra-cluster and intercluster degree and strengths, to μ_t_ = μ_w_ = *0.2*. Planted community sizes ranged from 5 to 50 nodes and were sampled from a power law with exponent τ_c_ = 1. This simulation was run 9 times for each value of μ_t_ = μ_w._ The Matlab code to generate LFR synthetic network is available at github.com/carlonicolini/lfrwmx. The function takes the parameters described above as inputs, and returns the equivalent to a weighted connectivity matrix that can be directly analyzed by community detection approaches.

### Simulation 2

**Figure 2:**
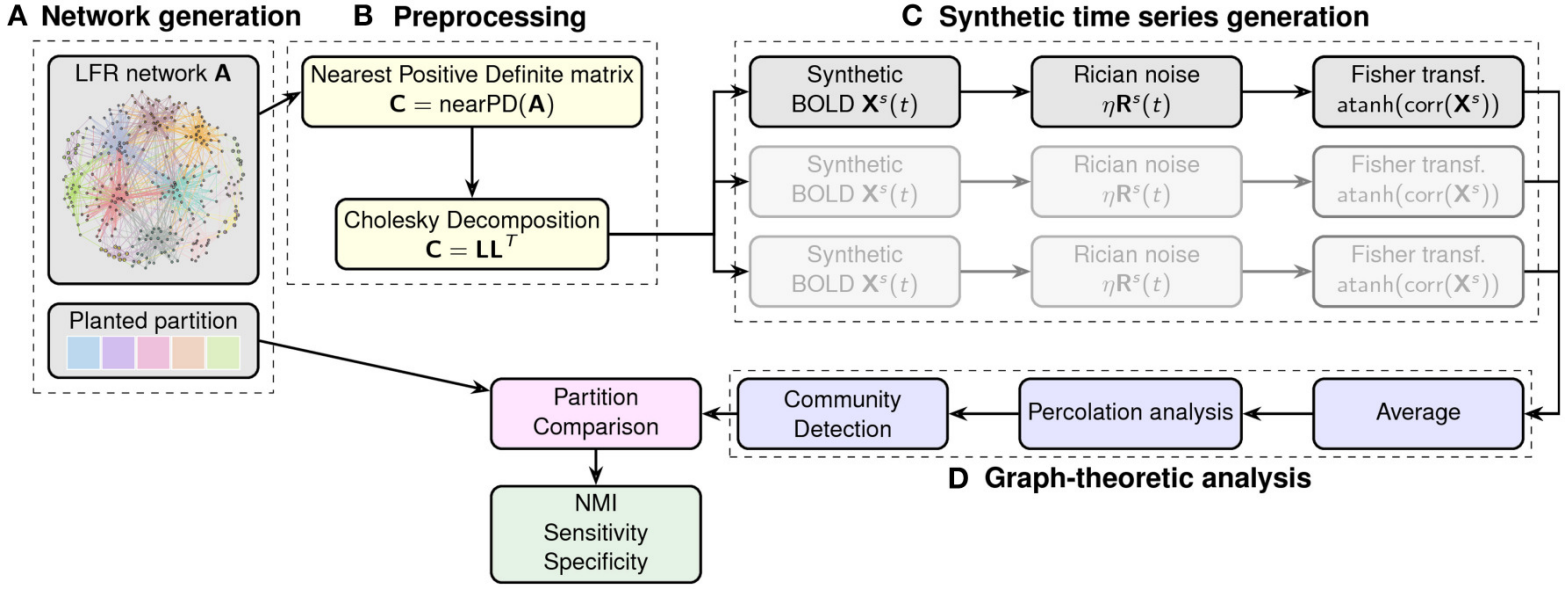
Flowchart of the generation and analysis of the synthetic datasets. In **(A)** a network with a pre-defined community structure is generated. The adjacency matrix is then processed in block **(B)** to obtain the nearest positive definite matrix for the Cholesky decomposition. This enables the generation of node-wise time-courses into which different levels of noise can be injected. The procedure is repeated multiple times to generate different instances (mimicking different subjects in the sample). Finally, correlation matrices are calculated for each instance (block **C**), and Fisher transformed to calculate the average adjacency matrix for analysis by community detection algorithms (block **D**). The resulting partitions are then compared with the original, planted one in terms of NMI.

This simulation makes use of the output matrix from the LFR function described above to generate artificial resting state fMRI datasets. The general idea is that, starting from an adjacency matrix with a given modular structure, we can generate time-courses for each of the nodes whose pairwise correlations reproduce the edge structure of the original matrix. Schematic of this procedure is shown in Figure 2.

To this end, we first calculate the closest positive-definite matrix *C* ∈ R^n×n^ from the adjacency matrix of the original LFR network (Higham, 1988). We then exploit the properties of the Cholesky decomposition and the techniques described in Nicolini et al. (2017) to calculate time-courses for the individual nodes. This approach makes it possible to generate correlated random variables, i.e., following the weights of our original connectivity matrix, by decomposing the closest positive definite matrix *C* ∈ R^n×n^ into the product of a lower triangular matrix *L* ∈ R^n×n^ and its transpose such that *C* = *LL*^*T*^. By multiplication of *L* with random standardized time series *X* ∈ R^n×m^ (our synthetic BOLD signals), we obtain new time series *Y* = *LX* whose covariance matrix is exactly *C* as one can verify that *C* = *E*(*YY*^*T*^) = *E*[(*LX*)(*LX*)^*T*^] = *E*[(*LX*)*X*^*T*^ *L*^*T*^] = *L E*[*XX*^*T*^]*L*^*T*^ = *L*^*T*^*IL* = *C*. The random time series were generated with 150 points and a base-line value set to 100. Both the random resting state time series *X* and the added noise were generated using the R package NeuRosim (Welvaert et al., 2011).

The generation of multiple sample of random time series simulates the effects of intersubject variability, and Rician-noise (Welvaert and Rosseel, 2013) is added to mimic fMRI resting state data. The definition of Signal-to-Noise (SNR) used in the rest of this paper is: SNR = $\overset{®}{S}/\sigma_{N}$ where $\overset{®}{S}$ is the average magnitude of the signal generated by NeuroSim and σ_*N*_ is the standard deviation of the noise (Krüger and Glover, 2001). An example of synthetic time-course is shown in the Supplementary Information section, Figure S1.

The last step results in 600 times series of 150 points with different levels of noise for each of the simulated subjects. Datasets were generated for populations of 20, 40, and 60 subjects and for a SNR equal to 35 and 70. The procedure has been run 5 times for each different parameter to produce different networks and datasets.

### Connectivity matrix

The connectivity matrix is the weighted matrix representing the links between two nodes. In Simulation 1, the matrix was generated directly by the LFR model. In Simulation 2, using the same approach as in fMRI experiment, we computed pairwise Pearson correlations between time-series from pairs of nodes in each dataset (subject), resulting in a matrix M of size N × N with N the number of nodes and with M(i,j) the correlation coefficient between the time series of the node i and the node j. Average group matrices were calculated by Fisher transformation and subsequent averaging of individual matrices.

### Sparsification and percolation threshold

Sparsification procedures are normally applied to remove weaker links, which are most affected by experimental noise (van den Heuvel and Fornito, 2014), and to reduce the density of the graph, thus making it computationally more tractable.

**Figure 3:**
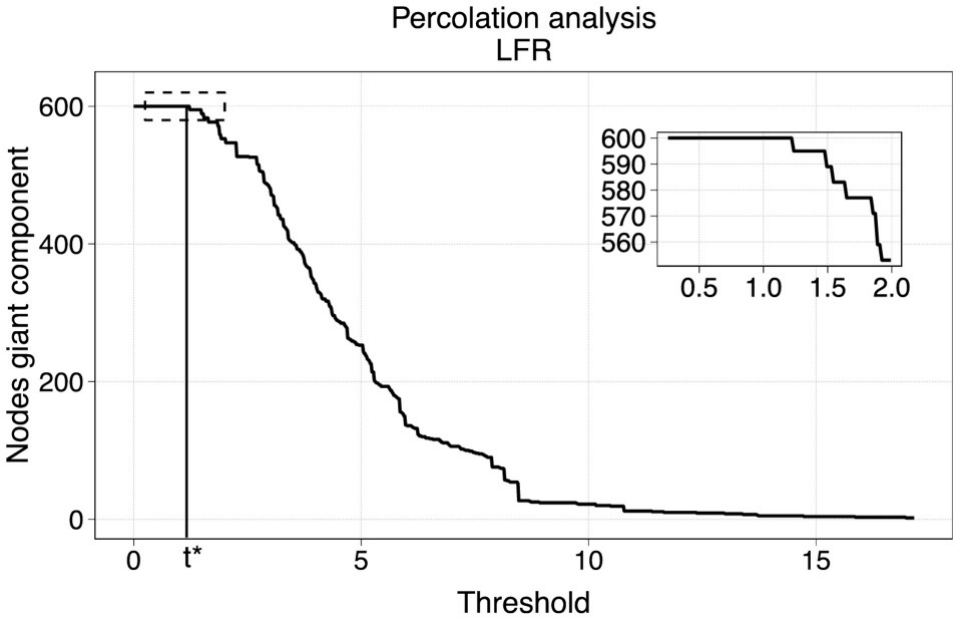
Percolation analysis for a LFR networks. The number of nodes in the giant component has a step-wise behavior with respect to the threshold. The percolation threshold value is t^*^.

The method of our choice for the sparsification was motivated by a model to describe phase transitions of connected subgraphs in random networks called percolation analysis (Callaway et al., 2000; Goerdt, 2001). We applied thresholds on the original network at different levels of edge weights, and identified the largest connected components of the thresholded graphs via breadth-first search (Leiserson et al., 2009). The critical point where the largest component starts breaking apart is identified as the percolation threshold at which the network's structure, is preserved while discarding potential effects of noise. Figure 3 represents an example of the size of the largest component with respect to the threshold in a benchmark LFR network.

### Community detection

To assess whether the efficacy of the sparsification procedure depends on the community detection approach, we applied three different methods, based on conceptually different principles that have been extensively applied to the analysis of resting state fMRI data. The first one, probably the most widely used, is Newman's modularity (Newman, 2006). We also tested InfoMap (Rosvall and Bergstrom, 2008) and Asymptotical Surprise (Nicolini and Bifone, 2016; Nicolini et al., 2017), as they have been shown to resolve community structures at a finer level than Newman's modularity, which is affected by a resolution limit that prevents detection of modules that are smaller than a scale determined by the size of the entire network.

Briefly, Newman's modularity seeks optimal partition by maximizing intra-cluster edge-density against that of a null model based on random edge rewiring. Optimization of this fitness function is typically performed using the Louvain method (Blondel et al., 2008), a greedy agglomerative clustering algorithm that works on hierarchical refinements of the network's partitions. Here we used the Louvain implementation available in the Brain Connectivity toolbox (Rubinov and Sporns, 2010).

The idea behind Infomap is the minimization, through a set of heuristics, of the description length (Rissanen, 1978) of a random walker defined on the network. For this study we used the Infomap implementation available in the igraph-0.7.1 package (Csárdi and Nepusz, 2006).

Finally, Asymptotical Surprise is a recently developed approach rooted in information theory that aims at maximizing the relative entropy between the observed intracluster density and the expected intracluster density, on the basis of the Erdos-Renyi null model (Traag et al., 2015). Surprise was recently shown to be quasi-resolution-limit free, and to provide improved means to resolve the modular structure of complex networks of brain functional connectivity (Nicolini and Bifone, 2016; Nicolini et al., 2017). Optimization of Asymptotical Surprise was carried out by means of PACO (PArtitioning Cost Optimization), an iterative agglomerative algorithm built on a variation of the Kruskal algorithm for minimum spanning trees (Nicolini and Bifone, 2016; Nicolini et al., 2017). We have shown that maximization of Asymptotical Surprise enables detection of communities of widely different sizes, thus making it possible to resolve differences in the modular organization of different networks representing functional connectivity in different subjects or experimental groups (Nicolini and Bifone, 2016). A Matlab toolbox including binary and weighted versions of Surprise optimization is available upon request at http://forms.iit.it/view.php?id=68447. An example of adjacency matrix for an LFR network with the node indexes reordered by membership and the modular partition demarcated by a red line is shown in the Supplementary Information section, Figure S2.

### Evaluation of retrieved partition

The advantage to know in advance the ground truth community is that we can quantify differences between the planted community and the extracted ones. Three coefficients were used to evaluate the results of the community detection methods at different levels of threshold of our synthetic networks. First, the Normalized Mutual Information (NMI) (Danon et al., 2005; Meilǎ, 2007), a measure of the similarity between structures is defined as:

(1) $$\begin{array}{r} NMI\left(A,B\right)=\frac{-2\sum_{i=1}^{C_{A}}\sum_{j=1}^{C_{B}}N_{ij}\log\left(\frac{N_{ij}N}{N_{i.}N_{.j}}\right)}{\sum_{i=1}^{C_{A}}N_{i.}\log\left(\frac{N_{i.}}{N}\right)+\sum_{j=1}^{C_{B}}N_{.j}\log\left(\frac{N_{.j}}{N}\right)} \end{array}$$

where *A* and *B* are the community structures of two networks, *C*_*A*_ and *C*_*B*_ are the number of community in partition *A* and *B* respectively, *N* the total number of nodes in the networks (which is the same in *A* and *B*) and *N*_*ij*_ is the overlap between *A*'s community *i* and *B*'s community *j*; i.e., the number of common nodes. Finally, *N*_*i*._ and *N*_.*j*_ are the total number of nodes in community *i* of *A* and *j* of *B* respectively. The NMI ranges from 0 to 1, where 0 indicates that the retrieved community structure does not convey information about the planted partition, and 1 when the two partitions correspond perfectly. Indeed, NMI = 0 corresponds to the situation of *N*_*ij*_ = 0, i.e., to a void intersection group between A's and B's communities, and NMI = 1 to complete identity.

In order to gain information about the origin of mismatches between planted and retrieved partitions, we also computed Sensitivity and Specificity, assessing the levels of false positives and false negatives incurred by the community detection algorithms. For each community we identified the biggest overlap between the ground truth and the retrieved modules to establish a correspondence between the partitions. Subsequently, we identified the nodes that were correctly assigned (true positives = TP) and wrongly assigned (false positives = FP) to a selected community. We also identified nodes that were correctly assigned (true negatives = TN) or erroneously assigned (false negative = FN) to a different community. These values were used to calculate Sensitivity and Specificity for each community:

(2) $$\begin{array}{r} Sensitivity=\frac{TP}{TP+FN} \end{array}$$

(3) $$\begin{array}{r} Specificity=\frac{TN}{TN+FP} \end{array}$$

and subsequently averaged over the partition. The values for Sensitivity and Specificity range from 0 to 1, with 1 denoting the perfect match.

### Benchmark resting state functional connectivity network

To illustrate the effects of threshold choice on the partition of resting state fMRI functional connectivity networks, we used a benchmark dataset described by Crossley et al. (2013). Detailed experimental and image processing procedures are described in the original paper, alongside with the ethical statements. In short, fMRI data were acquired from 27 healthy volunteers at 3 T. Gradient echo-planar imaging data were acquired for 5 min (TR = 2 s, TE = 13). Time series were extracted from 638 brain regions defined by a template also described in Crossley et al. (2013), and band-passed (0.01–0.1 Hz). Functional connectivity was defined as pairwise Pearson correlation at a subject's level, and group-level functional connectivity matrix was calculated by averaging individuals' matrices after Fisher- transform, We used BrainNetViewer as a tool for the visualization of the communities on brain templates.

## Results

### Simulation 1

**Figure 4:**
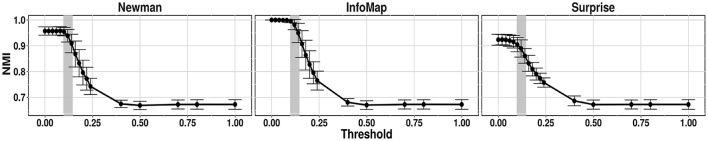
NMI between ground truth community structure and the results of the 3 community detection algorithms applied to an LFR networks (μ_t_ = μ_w_ = 0.2).

The benchmark created for this first test did not involve any variation coming from noise or subject variability. The community detections methods were applied directly to the matrix generated by the LFR function. Figure 4 shows the NMI calculated between the structure extracted by Newman modularity, InfoMap and Asymptotical Surprise, and the ground truth for μ_t_ = μ_w_ = 0.2, as a function of threshold.

The gray zone on the graphics indicates the range of sparsification thresholds obtained by percolation analysis calculated in different runs. These graphics demonstrate the deleterious effects of excessive removal of weak edges. In the case of noiseless networks, percolation analysis identifies the threshold corresponding to the departure from optimal performance of the community detection algorithm. This is in keeping with the fact that the percolation threshold is the minimum threshold value that preserves connectedness of the giant component. However, it should be noticed in this noiseless scenario all links correspond to true correlations, and no spurious edges are contemplated.

### Simulation 2

**Figure 5:**
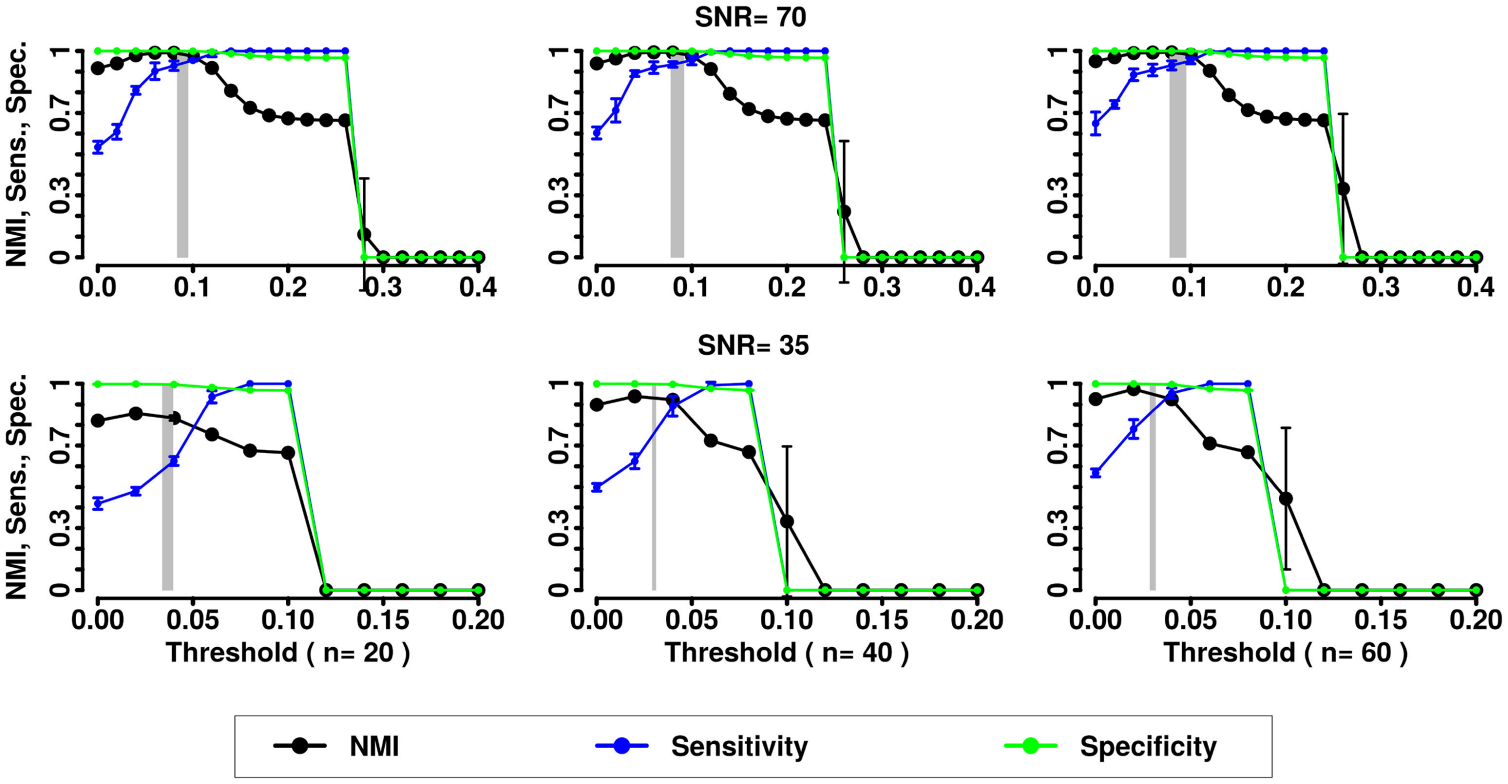
NMI (in black), Sensitivity (in blue), and Specificity (in green) of the Newman community detection algorithm applied to LFR networks (μ_t_ = μ_w_ = 0.2). Two different signal to noise ratio (SNR) are represented on the lines (top line SNR = 70, lower line SNR = 35), Number of subjects varies depending of the column (respectively from left to right 20, 40, and 60 subjects).

**Figure 6:**
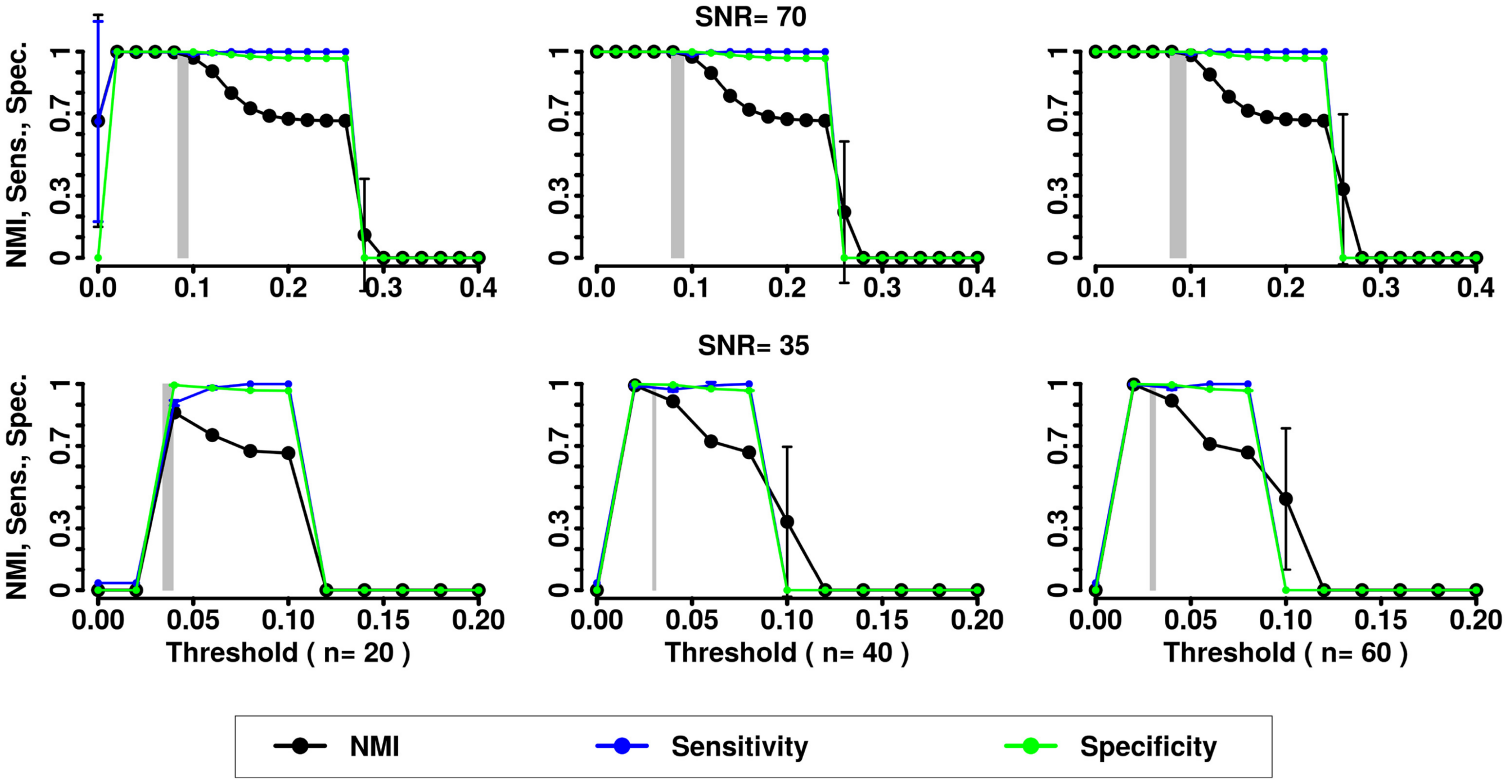
NMI (in black), Sensitivity (in blue), and Specificity (in green) of the InfoMap community detection algorithm applied to LFR networks (μ_t_ = μ_w_ = 0.2). Two different signal to noise ratio (SNR) are represented on the lines (top line SNR = 70, lower line SNR = 35), Number of subjects varies depending of the column (respectively from left to right 20, 40, and 60 subjects).

**Figure 7:**
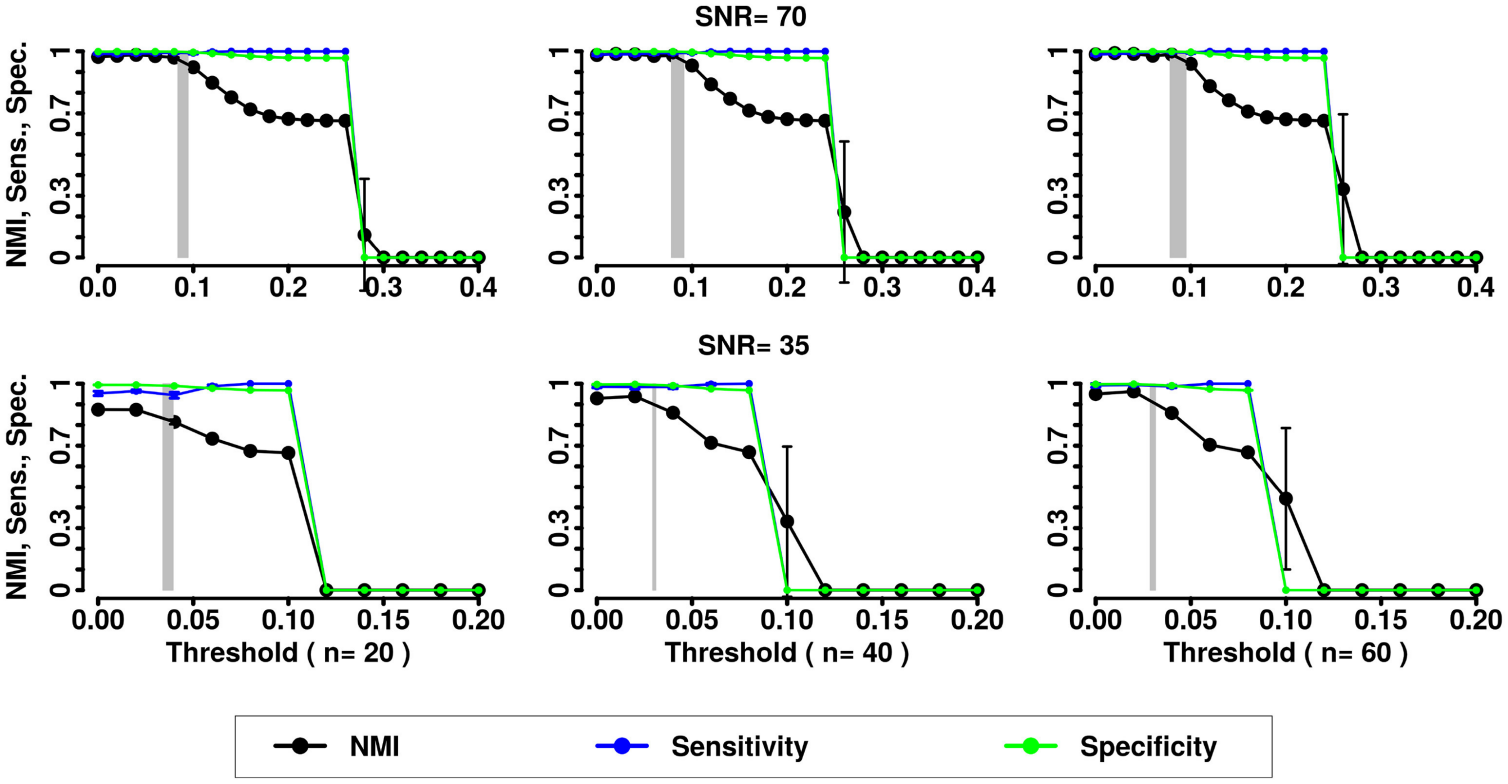
NMI (in black), Sensitivity (in blue), and Specificity (in green) of the Asymptotical Surprise community detection algorithm applied to LFR networks (μ_t_ = μ_w_ = 0.2). Two different signal to noise ratio (SNR) are represented on the lines (top line SNR = 70, lower line SNR = 35), Number of subjects varies depending of the column (respectively from left to right 20, 40, and 60 subjects).

In the second simulation we assessed the effects of noise and variability in the correlation structure of the networks. We computed NMI, the Sensitivity and Specificity for the partitions obtained by the 3 methods (Newman, InfoMap and Asymptotical Surprise) with the different SNRs and numbers of subjects (see Figures 5–7).

In the presence of variability, we observe a first increase in NMI for increasing threshold, followed by a subsequent drop. We interpret the first rise as a regime in which weak links are mostly determined by spurious correlations, and carry little information about the structure of the network. As threshold increases, removal of additional edges decreases the ability to retrieve the planted modular structure by removing structurally relevant correlations. This picture is confirmed by the observation that maxima in NMI correspond to simultaneously large values of Sensitivity and Specificity.

The percolation threshold values appear to consistently fall in the vicinity of maximum NMI for all three community detection methods. The general conclusion from these simulations is that percolation analysis detects a quasi-optimal value of sparsification threshold, thus enabling optimal detection of community structure in the presence of experimental noise and data variability.

### Effects of threshold in resting state brain networks

**Figure 8:**
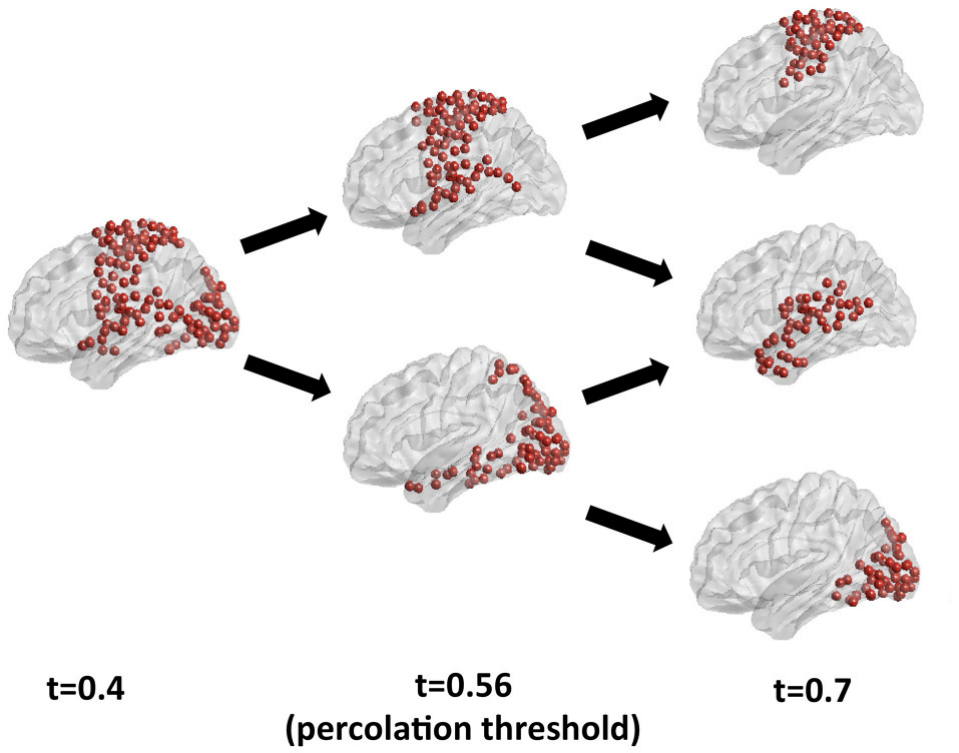
Analysis of the largest modules detected by Newman's community detection algorithm in a resting state functional connectivity network from the human brain for different thresholds. At percolation threshold (central panel) the sensorimotor and the visual modules are identified as separate communities, while they are merged into a widespread subnetwork for a threshold below percolation. For a higher threshold (right panel) fragmentation and reorganization of modules is apparent, with the emergence of a separate temporal module, and a break-up of dorsal sensorimotor and supramarginal and temporal cortices.

A ground-truth community structure for functional connectivity networks from the human brain remains to be established, as different community detection approaches retrieve different partitions depending on the characteristics of the fitness function and optimization algorithm. A discussion of the ultimately valid partition for functional connectivity networks and of the best algorithm for its retrieval is beyond the scope of this paper, which focuses on the information theoretical foundations of the choice of the optimal sparsification threshold. We have recently compared various community detection methods as applied to the study of human brain functional connectivity in Nicolini et al. (2017). Here, to illustrate the effects of the choice of threshold on community detection, we have applied Newman's modularity, probably the most established community detection algorithm in network neuroscience, to a benchmark resting state functional connectivity network for different threshold values. Figure 8 shows the largest module identified by Newman's modularity at a threshold below percolation (left panel), and its partitions for increasing thresholds. Below percolation threshold, a widely distributed subnetwork comprising sensorimotor, auditory and visual cortices is detected as a single community. At percolation threshold, this broad community breaks up into a sensorimotor module, which also includes the superior temporal gyrus, and an occipital module, including visual cortices as well as the ventral and dorsal visual streams. As the threshold is further increased, the algorithm retrieves a different modular organization, with a dorsal sensorimotor module separated from the supramarginal and temporal nodes, which merge with other temporal nodes to form an independent community. Hence, the effects of the choice of threshold are not limited to fragmentation of modules for increasing thresholds, but can also result in the mixing and merging of nodes from different communities into potentially spurious modules. This example further emphasizes the importance of a judicious choice of sparsification threshold. Our results in synthetic networks suggest that percolation analysis enables the identification of threshold that maximizes information on the network modular structure. A detailed description of the community structure of resting state functional connectivity brain networks at the percolation threshold is reported in Nicolini et al. (2017).

## Discussion

An open problem in the analysis of brain connectivity is the optimal choice of threshold when comparing different groups, e.g., patients and healthy controls in cross-sectional studies assessing the effects of disease on functional connectivity. Typically, identical sets of nodes are defined for the two groups, and the comparison is based on edge distribution and strength. Many studies tend to fix the same edge density in the connectivity graphs of the groups to be compared. Indeed, certain global topological parameters (e.g., global efficiency, Rubinov and Sporns, 2010) depend on edge density, and comparisons at constant density make it possible to assess differences related to the topological reorganization of links, rather than to their number and strength. On the other hand, constant edge density may bias group comparisons when graphs exhibit intrinsic differences in connectivity strength. By way of example, neuropsychiatric diseases like Schizophrenia and Autism have been associated with disruption and overall reduction of functional and structural connectivity. Imposing equal densities for graphs describing connectivity in patients and controls may lead to the inclusion of a greater number of weak, potentially spurious links in the group with weaker connectivity, and to the exclusion of important links in the group with stronger connectivity. A higher proportion of spurious connection results in a more random network topology, and intergroup differences may just reflect different levels of noise, rather than genuine topological differences (van den Heuvel and Fornito, 2014).

The present study may provide a strategy to overcome this problem. Indeed, community detection determines the membership of each node to a certain module. This is not dependent on overall edge density, but on the local balance between edges linking the node to other members of the same module, or to other nodes in different modules. The optimal sparsification threshold is the one that maximizes information about community structure, and is network-specific, as it depends on the structure and noisiness of each network. Hence, independent thresholding of the networks to be compared based on percolation analysis maximizes information about memberships in the two groups.

## Conclusion

In conclusion, we have explored the use of percolation analysis, a method based on statistical physics, to determine the sparsification threshold in synthetic networks endowed with a ground-truth modular structure, and topological features akin to those of real world networks like brain connectivity graphs. We find that the percolation threshold, i.e., the highest threshold that preserves connectedness of the giant component, corresponds to the maximum information that can be retrieved by various community detection algorithms on the planted modular structure in the presence of noise and intersubject variability. Intuitively, this threshold corresponds to the optimal balance between information lost by removing genuine edges and spurious correlations introduced by noise. These findings provide evidence of the existence of an optimal sparsification threshold, and a solid theoretical basis for its identification by means of a data driven method like percolation analysis.

## Author contributions

AB conceived the study, CB analyzed the dataset, CB, CN, and AB wrote the paper and reviewed the analysis. All authors reviewed the manuscript.

### Conflict of interest statement

The authors declare that the research was conducted in the absence of any commercial or financial relationships that could be construed as a potential conflict of interest.
