# Supplementary material for: Graph Analysis and Modularity of Brain Functional Connectivity Networks: Searching for the Optimal Threshold
Source: Front Neurosci. 2017 Aug 3;11:441. doi: 10.3389/fnins.2017.00441 (PMC5540956; doi:10.3389/fnins.2017.00441)
Supplement: Supplementary file 1 [file DataSheet1.docx]

**Supplementary Information**

**Graph analysis and modularity of brain functional connectivity networks: searching for the optimal sparsification threshold**

Cécile Bordier, Carlo Nicolini, Angelo Bifone

**Synthetic time-courses**


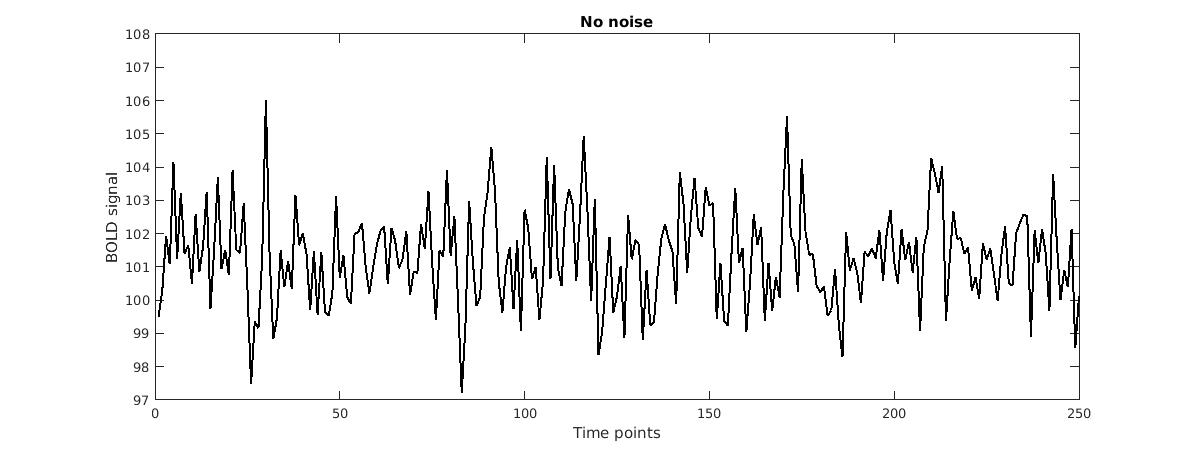


Fig. S1: Typical synthetic-BOLD time-course generated by NeuroSim. Time-courses in different voxels are designed to maintain the correlation structure of the original graph. Subsequently, noise in injected in the time-domain to mimic the effect of experimental noise. The amplitude of the noise is adjusted to obtain the desired Signal to Noise Ratio.

**Synthetic adjacency matrix**


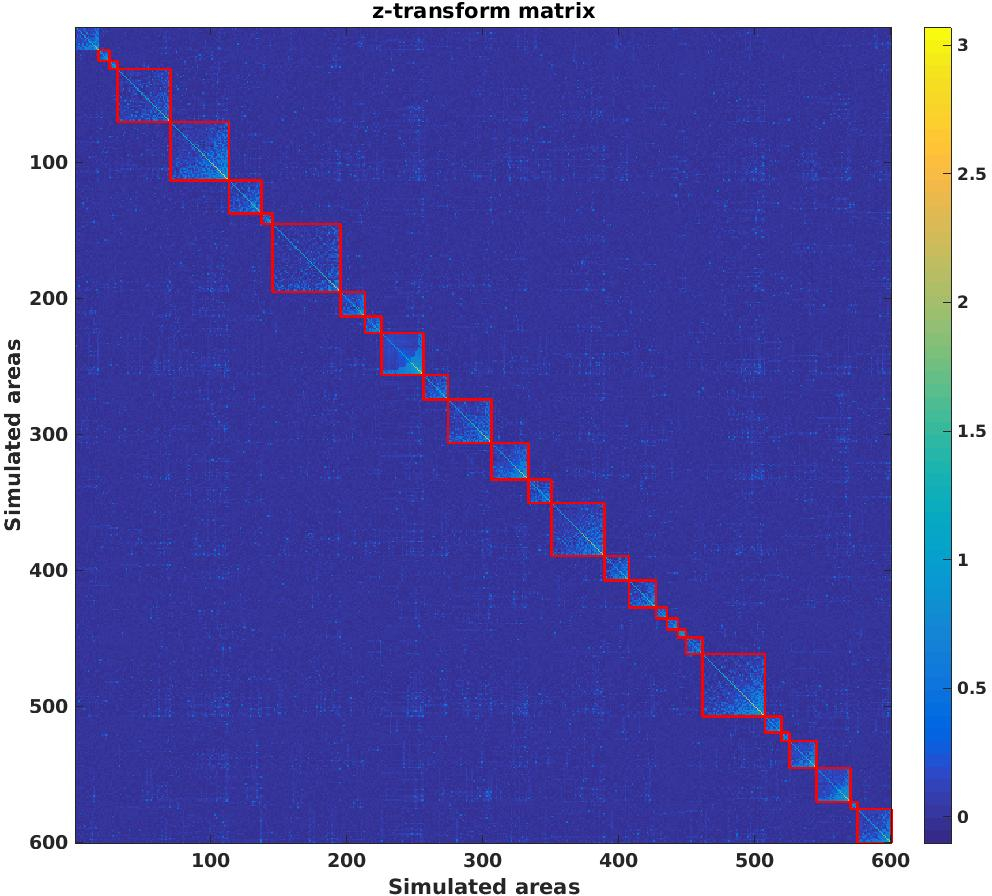


Figure S2: Example of group-level z-transformed adjacency matrix calculated with the procedure of fig. 2 in the main body of the Manuscript. Node indexes have been rearranged by membership and modules are demarcated by a red line.
